# ‘On paper, you’re normal’: narratives of unseen health needs among women who have had children removed from their care

**DOI:** 10.1093/pubmed/fdad137

**Published:** 2023-07-31

**Authors:** Claire Grant, Claire Powell, Georgia Philip, Ruth Blackburn, Rebecca Lacey, Jenny Woodman

**Affiliations:** Department of Epidemiology and Public Health, University College London, London, WC1E 7HB, UK; Great Ormond Street Institute of Child Health, University College London, London, WC1N 1EH, UK; Centre for Research on Children & Families, University of East Anglia, Norwich, NR4 7TJ, UK; Great Ormond Street Institute of Child Health, University College London, London, WC1N 1EH, UK; Department of Epidemiology and Public Health, University College London, London, WC1E 7HB, UK; Thomas Coram Research Unit, Social Research Institute, University College London, London, WC1H 0AA, UK

**Keywords:** child protection, lifecourse health, narrative analysis, public health

## Abstract

**Background:**

Mothers who have children removed from their care often have complex needs. These women have poor health outcomes and are dying earlier than their peers from preventable and amenable causes. Yet there is little known about how health care services might mitigate these risks. This study aimed to listen to the voices of women who had children removed from their care to understand their experiences of health and healthcare.

**Methods:**

We used a narrative approach to collect and analyse interview data with six mothers who had experienced child removal in England. Each participant was asked to reflect on their life and main health challenges.

**Results:**

Three narrative subplots were developed to consolidate experiences of unmet health need: (i) ‘on paper you’re normal’: narratives of complex need, (ii) ‘in my family, everyone had issues’: narratives of whole family need and (iii) ‘I’m still mummy, no matter where they are’: narratives of maternal identity and health.

**Conclusions:**

Findings highlight limitations within current systems of support, including a culture of distrust and women falling between the gaps of services. Women’s narratives illustrate opportunities for health intervention, especially immediately following child removal.

## Introduction

Child protective services have statutory power to intervene if they believe a child to be at risk of significant harm due to parental neglect, abuse or concerns around parenting capacity.^1^ Local authorities can introduce ‘child protection plans’, which strive to set out actions to keep the child safe, or in some cases, they may decide to take the child into care.^2^ Placing children in out-of-home care via legal care proceedings (‘child removal’) is considered the option of last resort. Despite this, rates of child removal in England are at the highest level ever recorded, with over 82 000 children looked after by local authorities.^3^ These trends disproportionately affect already disadvantaged families, such as children from minoritized ethnic groups^3^ and those living in poverty.^4^ In a recent independent review of children’s social care, there was a call to change how families are currently supported in child protection cases.^5^ In part, this advocates for addressing how services support the parents of these children.^6^

Women who have children removed from their care have complex health and social care needs, which can lead to child protection involvement and be exacerbated by child removal.^7^ Parental substance misuse, mental health need and developmental disabilities are frequent features in public care proceedings cases.^8^ Parents have often been exposed to high levels of trauma in their lives, with a recent review estimating that nearly one-third of parents experience clinically significant post-traumatic stress disorder symptoms.^9^ Child protective processes themselves add burden to families by increasing stress and threatening parental status.^10^ Although there is justification to protect the safety and wellbeing of at-risk children, child protection policies tend to neglect the historic and structural disadvantages these families face.^11^

Qualitative testimonies of birth mothers illustrate the immediate and enduring impact of child removal on women’s lives,^12^ including feelings of powerlessness, shame and further trauma.^13^^,^^14^ Women described feeling like ‘it was night all the time’, with acute grief that was often unsupported.^15^ It is estimated that one in four birth mothers return to the family court within 7 years of an initial set of care proceedings.^8^ These statistics indicate likely unmet needs of women who are left by public services after child protection involvement and caught in a cycle of recurrent child removal.^6^ In Canada and Sweden, national mortality data report that birth parents who have children removed are over three times more likely to die of a preventable cause compared to their biological siblings^16^ or to other parents.^17^

In a review of care proceeding court files in England, parental non-engagement with services was reported as the most frequent professional concern.^8^ Reasons for lack of engagement with health services can be complex, especially for those fearful of service involvement or experiencing multiple disadvantages, both of which are likely for birth mothers^12^^,^^18^ What’s more, there are known service gaps for women who have experienced child removal in England, with post-removal support dependent on local authority, charity or innovation funding.^19^ Understanding the experiences and needs of these women is essential for improving the development and delivery of public support services.

### Research question

This article aims to consider the experiences of health and healthcare for women who have their children removed by child protective services.

## Methods

Methods are reported in line with the Consolidated criteria for Reporting Qualitative research (COREQ).^20^

### Participants

Women were recruited through an organization who support birth mothers following child removal. This ensured participants were receiving comprehensive care and could access support following interviews if needed. Participants were required to speak and understand English. They could not be involved in ongoing care proceedings. Case workers at the organization offered study information to eligible women, who were invited to have a conversation with a researcher. Interviews took place online or in-person and lasted around 90 min. Six women were interviewed between March and June 2022 (see Table 1).

**Table 1:** Participant demographics

Pseudonym chosen by participant	Age range	Ethnicity	Children (*n*)	Child placement	Format
Ashley	20–29	White British	2	Foster Care	In-person
Erin	20–29	White British	5	Foster Care	In-person
Gina	30–39	Black British	1	Special Guardianship	Online
Kay	30–39	White British	2	Special Guardianship	Online
Sarah	20–29	White British	2	Adoption Order	In-person
Steph	40–49	White British	3	Foster Care	Online

### Approach

We used a combination of narrative approaches to collect and analyse interview data. Such approaches offer a means of collecting, examining and interpreting data with a storytelling form.^21^ Making sense of individual narratives can inform the lived realities of complex health and social care policy.^22^ These approaches consider multiple accounts of reality, as well as personal and societal influences that might impact on an individual’s understandings of events.^23^ We combined thematic narrative analysis, which is concerned with ‘what’ is being said, and personal narrative analysis, which places focus on how a story reveals truth about an individual’s sense of self and understanding of events.^24^ Narrative approaches can also mitigate risk of re-traumatization as participants hold the power as ‘storyteller’.^25^

### Analysis

Each interview was recorded, transcribed and used to develop a core story that encapsulated order and meaning of life events. Interviewer comments and participant pauses, noises and filler words were removed from the transcripts. Sections of text were reordered to construct stories chronologically. These were checked to ensure accuracy and legibility. Unlike other qualitative approaches, data were not broken down or ‘coded’, rather interpreted by researchers within the context of the whole story. Researchers developed a series of ‘subplots’ within each women’s story to consolidate threads of meaning, provide depth and add perspective to their health experiences. Where possible, these were discussed with participants to ensure researchers’ interpretations were reflective of women’s realities. In collaboration with a lived experience advisory group of mothers who had children removed from their care, the research team generated three overarching subplots to capture experiences across participants.

### Ethics

Ethical approval was obtained by the UCL Research Ethics Committee (reference: 21605/001).

## Results

Three subplots were constructed and named using participants own words: (i) ‘on paper, you’re normal’: narratives of complex need, (ii) ‘in my family, everyone has issues’: narratives of whole family need and (iii) ‘I’m still mummy, no matter where they are’: narratives of maternal identity and health.

### Subplot 1—‘On paper, you’re normal’: narratives of complex need

All women shared a range of conditions and needs which conveyed their overall health as poor. Most began by describing physical illness, including respiratory conditions, diabetes, chronic pain, bowel disorders, arthritis, osteoporosis, skin conditions, heart conditions, hypertension and blood clots. As stories evolved, women reflected on how physical conditions were often caused or worsened by trauma. All women described instances of being dismissed or inappropriately treated by clinicians who did not consider the wider context of their situation.

‘*I mean on paper, you’re normal. It’s just you suffer from psoriasis. When you go to the doctors, they just give you steroid creams. They didn’t offer support for the fact it’s a trauma-based condition. I’ve suffered from psoriasis for 20 years now and only at my last appointment did my dermatologist ask me about my life. For the first time they put together why I’m here and why it’s gotten so bad. You go to the doctors, and they give you just a few minutes. Nobody ever asked.*’*—**Gina***

Gina, Erin, Sarah and Kay described ineffective treatment plans which focused on pharmaceutical and self-help solutions. All women experienced an unsuccessful referral to a specialist mental health team. For example, Gina and Kay were told an addiction problem alone was not enough to see a psychologist. Erin described trying to lose weight to be eligible for her local eating disorder service. Steph had heard nothing back from an initial assessment over 2 years ago. Ashley’s experience of hallucinating was dismissed. After the death of her son, Sarah was not offered counselling.

‘*The doctor said depression pills would help. They don’t. The GP says they’re going to do something, but they’re just pill pushers. I don’t like pills. They make me feel funny. They made me feel like I was drunk, so I came off them. I gave up. I kept asking for a counsellor, but they just weren’t doing it. They’d give me links to these self-help pages, but I didn’t want that. They would give me leaflets too. A leaflet isn’t going to help me, talking to someone would.*’*—**Sarah***

### Subplot 2—‘In my family, everyone had issues’: narratives of whole family need

These complex health needs were endemic within families. Women described their needs as intrinsically linked to other family members. Kay, Ashley and Sarah grew up with siblings who had additional needs and parents with chronic conditions, developmental disabilities, mental illnesses or substance misuse disorders. Women reflected on feeling inadequately supported in their early years, often experiencing feelings of unwantedness. Sarah, Ashely and Erin spent time in foster care or with extended family for periods of time. Gina, Steph and Ashley had a parent die young, which meant they had to grow up fast and, at times, care for other family members. Each life story recalled multiple points of contact with health, education, justice and social care services, and most described a culture of distrust and scepticism toward public service support. Women described hiding their problems from others due to fear of judgement or repercussions, including from professionals. For some, substances were used to self-medicate unmet needs.

‘*I had a lot of traumas growing up as a child. My dad was very violent, and my brother had special needs. He’s bipolar, adult schizophrenic now and had ADHD. A lot of my parent’s attention was for him, and I was just on the back burner. I was too scared to ask for help or too scared to be judged. The only time I went to get help was probably as my kids were removed, because there’s always a stigma about it. I’ve been brought up to say what happens at home stays at home. You don’t tell anybody about your problems. That was really instilled in me as a child and even as an adult, we sort it out within ourselves.*’*—**Kay***

For Kay, Ashley, Steph and Gina, an abusive partner contributed toward their struggles. They felt that exposure to adversity in their early years normalized violence in their adult relationships. Kay and Ashley reflected on how a partner’s battle with addiction led to destructive and unsafe relationships which enabled their own struggles. Steph, Erin and Sarah faced additional challenges in managing care for their children who had physical disabilities, respiratory conditions and regular seizures. Steph described reaching out to children’s social care for help when caring for her child became unmanageable.

*‘When we first got together, he had depression, but it turned into a lot more. He would have very, very dark mood swings. I think he’s since been diagnosed with borderline personality disorder and schizoaffective disorder. He was hard work, and he was abusive. Then my son was born about four weeks early. He obviously needed some special care and stuff. It was a really difficult time.’—**Steph***

### Subplot 3—‘I’m still mummy no matter where they are’: narratives of maternal identity and health

Women’s health needs were exacerbated by child removal. Most experienced acute grief in the immediate stages following child removal but had inconsistent or inadequate support from services. For Kay, Erin, Ashely, Gina and Steph, being a mother was a fundamental part of their identity and something which persisted beyond their children being removed. Women shared how no longer being viewed as ‘mothers’ by public services impacted on their access to care.

‘*Do you know when my children were removed, local authorities did not contact me at all. There was no contact. I’m not being funny. Like I could have killed myself and I don’t think they would have known. That’s how bad it is. Keeping in mind that they are looked after in care children, which means they have a duty of making sure that I’ve got this support when my children are removed. I didn’t have nothing. Nothing whatsoever. All they will contact me for was parenting assessments for when we went back to court.*’*—**Erin***

Most women described fear of child protection involvement as a barrier to accessing support for their mental health and addiction. Kay and Erin reflected on their children being removed as a catalyst for seeking help as they felt they had nothing left to lose. This was not completely consistent across all interviews: Sarah described never really feeling like a mother to her children and viewed their out-of-home placement as a positive solution. Women’s sense of motherhood influenced how they worked with services and how they made sense of their needs.

‘*I’ve always found the children to be grounding. I can’t leave them. I’m not gonna leave them. So even in my darkest moments, I think of the children. It doesn’t take away the anxiety or depression, but it gives me reason to keep going, to fight. They’ve always been the best things in my life. Even now, when none of them are living at home, they’re still everything. A few months I was prepared to end it all, but I said to myself*, “*what are you going to tell the kids?*” *I tried writing this letter to them and it kind of brought me back. I just thought, I can never leave them.*’*—**Ashley***

## Discussion

### Main findings of this study

Among birth mothers who had children removed from their care by child protective services, poor health was common and often normalized. In keeping with previous research, our findings suggest that health inequity was compounded by adverse experiences and disadvantages throughout women’s lives.^26^ There is a clear cycle of deprivation and trauma, as mothers describe growing up in homes with complex need and having these experiences continue into their own families as they become parents. Support services should consider ‘whole family’ need to interrupt intergenerational trends, including supporting current children, birth fathers and any potential future children they may have.^7^^,^^27^

Narratives illustrate that women’s health needs are often unrecognized by professionals, with services providing treatment for symptoms which ‘on paper’ seem manageable but are often indicative of complex unseen need. In part, these narratives depict a culture where women fear judgment or repercussions from services and are conditioned to hide problems as a result. Narratives also reveal truths around experiences of siloed healthcare provision which can leave women experiencing multiple adversities falling between the gaps of services. This is substantiated by the finding that all birth mothers interviewed were deemed ineligible for specialist mental health support, despite most women facing severe mental health challenges. Findings strongly suggest that multi-disciplinary support is needed to consider the wider context of women’s health which might contribute toward preventable illness and mortality among birth mothers.^17^^,^^28^ Although there are opportunities for intervention before child protection involvement, there is clear justification for accelerated support for birth mothers following child removal.^6^

Currently, there is no statutory requirement to support parents once a child is taken into care in England, with a ‘postcode lottery’ of local authority provision.^19^ Entitlement to support may also vary depending on legal status, for example if a child is placed under an adoption order or special guardianship order. Women often stop being viewed as mothers by services after their children are removed, as support from children’s social care and perinatal services falls away.^6^^,^^26^ This can leave women without appropriate support in a time of acute mental health need, where they are left facing the challenges which led to child protection involvement and the compounding impact of child removal.^7^^,^^12^

Women’s narratives illustrate the power of maternal identity as a motivating factor for change and growth. Motherhood can provide a sense of hope and purpose, yet following child removal there are few opportunities to navigate loss and renegotiate maternal identity.^12^ Health services supporting women following child removal have a duty to consider and acknowledge the role of being a mother and its impact on health, even when children are no longer in women’s care.^29^

### What is already known on this topic

It is well documented that the health of mothers who have children removed by child protective services is poor.^7^ We also know that health needs can be worsened by child removal, with deteriorating mental health,^15^ increasing risky behaviours^8^ and avoidable mortality.^17^

### What this study adds

This study demonstrates that many health needs are unseen and unmet by healthcare. Findings indicate a need for services to address underlying adversities and trauma that manifest in services as ‘normal’ health need. Women described a tendency of hiding needs from professionals due to an ingrained distrust of services, as well as being desensitized to poor health. Access to compassionate and multidisciplinary care should be available for mothers, especially following child removal.^19^ One suggested approach is the implementation of an advocacy caseworker model. Similar approaches have been trialed for survivors of domestic abuse^30^ and women who are refugees or asylum seekers.^31^ In principle, this provides support from trained individuals who can help women make plans, access resources and enter appropriate services.^32^ ‘Pause’ offers a national example of how mothers involved in recurrent care proceedings might be supported by a case-worker model and be supported into relevant services.^33^ Other examples of locally developed services are demonstrated to be feasible and largely acceptable by mothers and service providers across England.^19^^,^^33^

### Limitations of this study

We only spoke to women who had children removed from their care and so these data do not capture how healthcare services might interrupt in child protection intervention through whole family, parent or child support.^34^ We also recruited through an organization providing comprehensive support to mothers and so do not hear the voices of women without support. Further research should consider involving fathers to understand experiences of both parents.^35^

## Conclusion

Findings highlight limitations within current systems of support, including a culture of distrust and women falling between the gaps of services. Narratives illustrate opportunities for health intervention to address unmet need for mothers, especially following child removal.

## Notes

**Claire Grant**, PhD candidate

**Claire Powell**, Senior Research Fellow

**Georgia Philip**, Lecturer

**Ruth Blackburn**, Senior Research Fellow

**Rebecca Lacey**, Associate Professor

**Jenny Woodman**, Associate Professor
